# A modified endoscopic full-thickness resection for early colorectal cancer: An expanded application based on super minimally invasive surgery technology

**DOI:** 10.1055/a-2410-3269

**Published:** 2024-10-25

**Authors:** Xiaoqing Zhou, Bo Ning, Qianqian Chen, Enqiang Linghu

**Affiliations:** 1 Department of Gastroenterology, The First Medical Center of PLA General Hospital, Beijing, China; 2 481107 Nankai University School of Medicine, Tianjin, China

With the development of reliable endoscopic closure techniques and tools, endoscopic full-thickness resection (EFTR) is emerging as a therapeutic option for the treatment of some challenging lesions that cannot be adequately and/or safely treated with endoscopic mucosal resection (EMR) or endoscopic submucosal dissection (ESD) 1 . In order to solve the problem that traditional EFTR is prone to complications such as pneumoperitoneum, our team invented a modified endoscopic full-thickness resection, taking the full-thickness resection and closure of the defect at the same time until the lesion is completely removed, as has been used to resect large gastrointestinal stromal tumors 2 . Due to the complexity of the structure, the development of this technique in colonic lesions is more challenging. This time, our team applied this modified EFTR to the treatment of early colon cancer successfully ( Video 1 ).

A modified endoscopic full-thickness resection for early colorectal cancer.Video 1

First, the lesion was fully exposed and the margin of the lesion was marked by argon plasma coagulation (APC) ( Fig. 1 **a,b** ). Second, a submucosal injection was performed to separate the mucosal layer surrounding the lesion from the submucosa ( Fig. 1 **c** ). Third, the edge of the lesion was cut with a DualKnife, and the lesion was pulled to the contralateral side with a figure-8 ring ( Fig. 1 **d,e** ). Subsequently, the lesion was gradually removed by a DualKnife, IT knife, and triangle knife. Full-thickness resection was performed to resect the lesion and tissue clamps were used to close the incision during resection ( Fig. 1 **f** ). After complete resection, tissue clamps were applied again to seal the mucosa ( Fig. 1 **g** ).

**Fig. 1:**
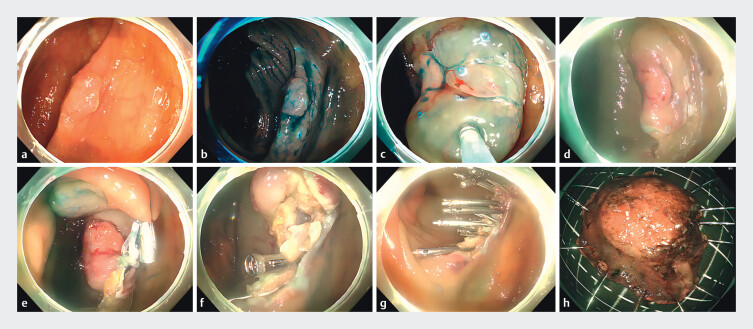
The procedure of modified endoscopic full-thickness resection for early colorectal cancer. **a** The feature of the early colorectal cancer was revealed under white light endoscopy. **b** The margin of the lesion was marked by argon plasma coagulation and then indigo carmine was sprayed to further define the scope. **c** A submucosal injection was performed to separate the mucosal layer surrounding the lesion from the submucosa. **d** A circumferential submucosal incision was performed to adequately expose the lesion. **e** The lesion was pulled to the contralateral side with a figure-8 ring. **f** A full-thickness resection was performed to resect the lesion. Clips were used to close the submucosal layer of the defect at the same time. **g** The lesion was completely removed and the submucosal layer of the defect was sutured. **h** The morphology of the lesion was shown under narrow-band imaging.

This study is the first to use a modified EFTR technique for the treatment of early colon cancer, enabling full resection of the lesion while reducing gas-related complications. In summary, the value of this technique is further extended to clinical applications, but robust prospective studies are still needed to obtain more reliable statistical evidence.

Endoscopy_UCTN_Code_TTT_1AQ_2AD_3AF

## Notes

Endoscopy E-Videos https://eref.thieme.de/e-videos
*E-Videos* is an open access online section of the journal *Endoscopy* , reporting on interesting cases and new techniques in gastroenterological endoscopy. All papers include a high-quality video and are published with a Creative Commons CC-BY license. Endoscopy E-Videos qualify for HINARI discounts and waivers and eligibility is automatically checked during the submission process. We grant 100% waivers to articles whose corresponding authors are based in Group A countries and 50% waivers to those who are based in Group B countries as classified by Research4Life (see: https://www.research4life.org/access/eligibility/ ). This section has its own submission website at https://mc.manuscriptcentral.com/e-videos .
